# Report of One-Year Prospective Surveillance of SARS-CoV-2 in Dogs and Cats in France with Various Exposure Risks: Confirmation of a Low Prevalence of Shedding, Detection and Complete Sequencing of an Alpha Variant in a Cat

**DOI:** 10.3390/v13091759

**Published:** 2021-09-03

**Authors:** Emilie Krafft, Solène Denolly, Bertrand Boson, Sophie Angelloz-Pessey, Sophie Levaltier, Nicolas Nesi, Sandrine Corbet, Bryce Leterrier, Matthieu Fritz, Eric M. Leroy, Meriadeg Ar Gouilh, François-Loïc Cosset, Angeli Kodjo, Vincent Legros

**Affiliations:** 1 Campus Vétérinaire de Lyon, VetAgro Sup, Université de Lyon, 69280 Marcy-l’Etoile, France; emilie.krafft@vetagro-sup.fr (E.K.); sophie.angelloz-pessey@vetagro-sup.fr (S.A.-P.); angeli.kodjo@vetagro-sup.fr (A.K.); 2 CIRI—Centre International de Recherche en Infectiologie, Team EVIR, Université de Lyon, Université Claude Bernard Lyon 1, Inserm, U111, CNRS, UMR5308, ENS Lyon, 69364 Lyon, France; solene.denolly@ens-lyon.fr (S.D.); bertrand.boson@ens-lyon.fr (B.B.); 3 Groupe de Recherche sur l’Adaptation Microbienne (GRAM 2.0), Normandie Université, UNICAEN, UNIROUEN, EA2656, 14032 Caen, France; sophie.levaltier@gmail.com (S.L.); nicolas.nesi@unicaen.fr (N.N.); bryce.leterrier@univ-rouen.fr (B.L.); meriadeg.legouil@normandie-univ.fr (M.A.G.); 4 Laboratoire de Virologie, Centre Hospitalo-Universitaire, 14033 Caen, France; corbet-s@chu-caen.fr; 5 Institut de Recherche pour le Développement (IRD), Maladies Infectieuses et Vecteurs, Ecologie, Génétique, Evolution et Contrôle (MIVEGEC) (Université de Montpellier—IRD 224–CNRS 5290), 34394 Montpellier, France; matthieu.fritz@ird.fr (M.F.); eric.leroy@ird.fr (E.M.L.)

**Keywords:** SARS-CoV-2, variants, prevalence, sequencing, zoonosis

## Abstract

Despite the probable zoonotic origin of SARS-CoV-2, only limited research efforts have been made to understand the role of companion animals in SARS-CoV-2 epidemiology. According to recent serological prevalence studies, human-to-companion animal transmission is quite frequent, which led us to consider that the risk of SARS-CoV-2 transmission from animal to human, albeit negligible in the present context, may have been underestimated. In this study, we provide the results of a prospective survey that was conducted to evaluate the SARS-CoV-2 isolation rate by qRT-PCR in dogs and cats with different exposure risks and clinical statuses. From April 2020 to April 2021, we analyzed 367 samples and investigated the presence of SARS-CoV-2 RNA using qRT-PCR. Only four animals tested positive, all of them being cats. Three cats were asymptomatic and one presented a coryza-like syndrome. We describe in detail the infection in two cats and the associated clinical characteristics. Importantly, we obtained SARS-CoV-2 genomes from one infected animal and characterized them as Alpha variants. This represents the first identification of the SARS-CoV-2 Alpha variant in an infected animal in France.

## 1. Introduction

Despite the probable zoonotic origin of SARS-CoV-2, and the demonstration that companion animals can be infected both in the laboratory [1] and in natural settings [2], as previously reported for SARS-CoV-1 during the 2003 outbreak [3], only a few reports presenting the clinical signs associated with SARS-CoV-2 infection in cats or dogs have been published so far. Indeed, on 27 July 2021, only 97 cats and 85 dogs were confirmed positive in the United States by the USDA [4]. Recent serological studies demonstrate a relatively high seroprevalence of the infection in dogs and cats living in close contact with infected humans [5,6,7,8,9], indicating frequent human-to-companion animal transmission. Companion animal infections are probably underrated, due to the lack of clinical signs in most infected animals. Indeed, SARS-CoV-2 infection seems to be predominantly asymptomatic, as highlighted by several experimental infections [10,11]. Additionally, cat-to-cat transmission has been demonstrated in laboratory settings [1,11], but cat-to-human or dog-to-human transmission has never been described. Importantly, those cat-to-cat transmissions were possible even between asymptomatic cats [11]. There are more than 100 million cats and 90 million dogs in Europe [12], with a relatively high seroprevalence revealed by a rate of seroconversion starting from 0.2% and reaching 14.69% in the general companion animal population, and reaching from 21–53% in those living in SARS-CoV-2-infected households [6,13,14,15,16,17,18,19,20,21]. Altogether, these observations led us to consider that the role of pets, albeit limited in the global SARS-CoV-2 epidemiology, might be more significant when humans come in close contact with animals living in SARS-CoV-2-positive households. Quantifying the virus isolation rate in dogs and cats with variable exposure risks would help to evaluate the risk of contamination for handlers, including owners, and caregivers, such as veterinarians. Moreover, the emergence of new variants prompts further investigations. The first SARS-CoV-2 variant was identified in February 2020, with an S-D614G substitution leading to the emergence of a more transmissible virus that rapidly became predominant. More variants were observed in the last trimester of 2020, including the B1.1.7 variant with seven substitutions and two deletions in the S protein, the B.1.351 variant, the P1 variant, and the B.1.617.2 variant, first identified in the United Kingdom, South Africa, Brazil, and India, and now defined as the Alpha, Beta, Gamma, and Delta variants, respectively [22]. These four variants are classified as Variants of Concern (VOCs), a classification in which variants with increased transmissibility, higher pathogenicity, or potential immune escape are grouped. Today, whether those variants are associated with higher transmissibility or more severe clinical signs during companion animal infection is not clear, but the recent discovery of symptomatic SARS-CoV-2 infection in pets with severe myocarditis in England during the emergence of the Alpha variant is concerning [23]. Thus, it is important to identify and characterize infection in the companion animal population, and to monitor the sequence of the virus to identify the potential mutations associated with infection in animals. In this report, we present a prospective survey that was conducted to evaluate the SARS-CoV-2 isolation rate by qRT-PCR in dogs and cats with different exposure risks and clinical statuses. We also describe in detail the infection in two cats and the associated clinical characteristics. Importantly, we obtained SARS-CoV-2 genomes from one infected animal and characterized them as Alpha variants. This represents the first identification of the SARS-CoV-2 Alpha variant in an infected animal in France.

## 2. Materials and Methods

### 2.1. Samplings

A total of 367 oropharyngeal swabs were obtained. Selected animals were sampled (oropharynx and fecal) with sterile viscose swabs (Citoswab, Wellkang, Dover, UK) and samples were immediately plugged into virus preservation liquid (eNAT, Copan, Brescia, Italy). Samples were then transferred to the laboratory (the day of sampling or, maximum, the day after sampling) for immediate processing upon arrival.

### 2.2. Viral RNA Extraction

The KingFisher Flex 96 (Thermo Fisher Scientific, Waltham, MA, USA), and the Auto Pure 96 (Allsheng Instruments CO, Hangzhou, China) automated extraction instruments were used to extract viral RNA from swabs, using the BioExtract Premium Mag (Biosellal, Dardilly, France) according to the manufacturer’s instructions. Viral RNA was eluted with 100 µL buffer and used for the qRT-PCR assay.

### 2.3. SARS-CoV-2 RNA Detection Using qRT-PCR

The Bio-T kit TriStar COVID-19 (Biosellal, France), which targets the envelope gene (E) of the Sarbecovirus, and RNA-dependent RNA polymerase (RdRp) in the Orf1ab gene, were used for SARS-CoV-2 RNA detection, according to the manufacturer’s instructions, using 5 μL of extracted RNA. The AriaMx Real-Time PCR Thermal Cycler System (Agilent Technologies, Santa Clara, CA, USA) was used for amplification. The conditions consisted of 1 cycle of 20 min at 50 °C, 1 min at 95 °C, followed by 40 cycles of 10 s at 95 °C, and 45 s at 60 °C. The results were analyzed using Agilent Aria 1.6 software, in which a cycle threshold value (Ct-value)  <  40 for all target genes was defined as a positive result.

Positive samples were confirmed in the virology unit of the University Hospital of Caen using SARS-CoV-2-standardized real-time multiplex one-step reverse-transcription PCR protocol targeting Orf1, S and N genes. RNA extracts were submitted to the 3-AllplexTM 2019-nCoV assay (Seegene, Seoul, South Korea), using the manufacturer’s instructions for the CFX-96 RT System C1000 Thermal Cycler (Biorad, Hercules, CA, USA), and the SARS-CoV-2 Viewer for Real-Time, instrument version 3.19.003.010 (Seegene, Seoul, Korea).

### 2.4. Sequencing

Sequencing was performed using an in-house protocol developed at the virology laboratory of the University Hospital of Caen for the routine sequencing of SARS-CoV-2. This protocol is based on nanopore library preparation adapted to two pools of PCR-amplified products using ARTIC primer scheme version 3 which produces ~400 bp of overlapping amplicons over the SARS-CoV-2 genome.

### 2.5. Amplification of Genomic Material

Briefly, after the random reverse transcription of 8 µL of RNA using the SuperScript Vilo enzyme (Life Technologies, Foster City, CA, USA), we performed multiplex PCRs in 2 pools using ARTIC V3 primers and 35 cycles of amplification with a high-fidelity polymerase.

### 2.6. ONT Library Preparation and MinION Sequencing 

Libraries were prepared following the Oxford Nanopore Technologies (ONT, Oxford, UK) protocol for the native barcoding of genomic DNA sequencing involving the barcode ligation sequencing kit SQK-LSK109 and the EXP-NBD104/114 Native Barcoding kit. Sequencing libraries were constructed, and sequencing was performed according to the manufacturer’s instructions, as briefly described below. Amplicons were pooled, bead-purified AMPure XP beads (Beckman Coulter, High Wycombe, UK), and normalized to 1 µg before end repair with NEBNext Ultra II End Repair (E7546S, New England Biolabs, Ipswich, MA, USA) and subsequent library preparation. End-prepared amplicons were ligated with native barcode adapters NBD04 using Blunt/TA Ligase Master Mix (M0367S, New England Biolabs, Ipswich, MA, USA) and then purified with AMPure XP beads (1× ratio). The two samples were then pooled to produce a 60 μL equimass pool used for adapter ligation with 10 µL of the Adapter Mix II (AMII), 20 µL of the NEBNext Quick Ligation Reaction, and 10 µL of Quick T4 DNA ligase. After 15 min of room temperature incubation, the material was bead-purified using provided SFB and EB before the final library was mixed and loaded onto an R9.4 flow cell (FLO-MIN106, Oxford Nanopore Technologies) and the run was performed on a MinION Mk1B device (ONT) in order to obtain 1 Gb of raw data per sample in 1.5 h.

### 2.7. Genome Assembly and Phylogenetic Inference

Sequencing data were basecalled with a quality score of 6 and subsequently demultiplexed using Guppy GPU basecaller and barcoder (ONT). Raw reads were trimmed of indexes, primers, and adapters using Porechop [24]. Cleaned reads were then mapped against a custom reference of SARS-CoV-2 genome comprising 4 Chinese and 70 early French sequences using Bowtie2 [25] and Minimap2 [26]. Finally, a consensus genome sequence based on mapped reads was generated with bcftools consensus [27]. SARS-CoV-2 sequences were deposited on GISAID under numbers EPI_ISL_3838696 and EPI_ISL_3857106. Consensus sequences generated were aligned with 760 SARS-CoV-2 genomes representing the diversity of major lineages circulating in France from March 2020 to March 2021 using MAFFT [28]. Alignment was then visually inspected in BioEdit 7.0.5 [29]. We inferred the phylogenetic tree using maximum likelihood (ML) with IQ-TREE 2 [30]. 

### 2.8. Serological Testing

#### 2.8.1. Microsphere Immunoassay

Serum samples from cats and dogs were analyzed using a multiplex microsphere immunoassay (MIA). The MIA procedure was performed as described previously [5]. Briefly, 10 µg of nucleoprotein (N), receptor binding domain (RBD), and trimeric spike (tri-S) was used to capture specific antibodies in samples. According to the manufacturer’s instructions, distinct MagPlex microsphere sets (Luminex Corp, Austin, TX, USA) were coupled to viral antigens with the amine coupling kit (Bio- Rad Laboratories). As a control, we used a microsphere set coupled with recombinant human protein (O6-methylguanine DNA methyltransferase). Microsphere mixtures were incubated in the dark on an orbital shaker with serum samples (1:400), biotinylated protein A and biotinylated protein G (4 μg/mL each) (Thermo Fisher Scientific, Illkirch, France), and Streptavidin-R-Phycoerythrin (4 μg/mL) (Life Technologies, Illkirch France), and measures were obtained with a Luminex 200 instrument (Luminex Corp, Austin, TX, USA). For each bead set, a minimum of 100 events were read and are displayed as the median fluorescence intensity (MFI). To set the seropositivity, 29 dogs’ and 30 cats’ sera sampled before 2019 were measured as the negative control. Cut-off values were set as three standard deviations above the mean MFI of the negative controls. MIA specificity was 96.7% for dogs and 96.4% for cats for each antigen.

#### 2.8.2. Neutralization Activity Measurement

The neutralizing activity in cat and dog sera was measured using a pseudoparticle neutralization system. Briefly, an MLV-based pseudoparticle carrying a GFP reporter pseudotyped with SARS-CoV-2 spike (SARS-CoV-2pp) was used. Each sample was processed as previously described [5]. Briefly, a sample of ~1 × 10^3^ pseudoparticles was incubated with a serial dilution of serum for 1 h at 37 °C before infection of the Vero-E6R cells (ATCC CRL-1586). The percentage of GFP-positive cells was compared to cells infected with SARS-CoV-2pp incubated without serum to set the percentage of neutralization. Sera collected in France before 2019 were used as negative controls, together with a commercial anti-SARS-CoV-2 RBD (Sino Biological, Beijing, China) as a positive control.

## 3. Results

To better understand the risk of SARS-CoV-2 transmission from companion animals to owners or veterinarians, we set up a prospective survey to identify dogs and cats with SARS-CoV-2 by detecting RNA from oropharyngeal and fecal swabs. 

### 3.1. Prospective Survey

The survey was conducted from April 2020 to April 2021 and samples were obtained after informed consent from their owners. Animals were classified into two groups: asymptomatic (or with symptoms unrelated to SARS-CoV-2 infection) or symptomatic (with clinical signs consistent with SARS-CoV-2 infection). Asymptomatic animals (Group A) were recruited from animals visiting VetAgro Sup veterinary hospital and divided into two groups depending on their exposure status. Group A1 was recruited from animals owned by people with either suspected or confirmed (qRT-PCR positive) SARS-CoV-2 infection. Dogs and cats presented to our hospital with no or unknown household exposure were sampled randomly to form group A2.

Animals with clinical signs compatible with previous descriptions of cases of symptomatic feline and canine SARS-CoV-2 infection (Group S) were recruited from the animals admitted to our hospital or collaborating private practices. In order to be included in Group S, cases had to fulfill the two following criteria: (1) show clinical signs consistent with COVID-19, such as respiratory problems, sneezing, nasal discharge, cough, and dyspnea, with or without fever, or gastrointestinal signs or a clinical suspicion of myocarditis; and (2) be owned by people with either suspected or confirmed (qRT-PCR positive) recent SARS-CoV-2 infection. Oropharyngeal swabs were obtained from all animals and rectal or feces swabbing was also performed punctually. 

**Figure 1:**
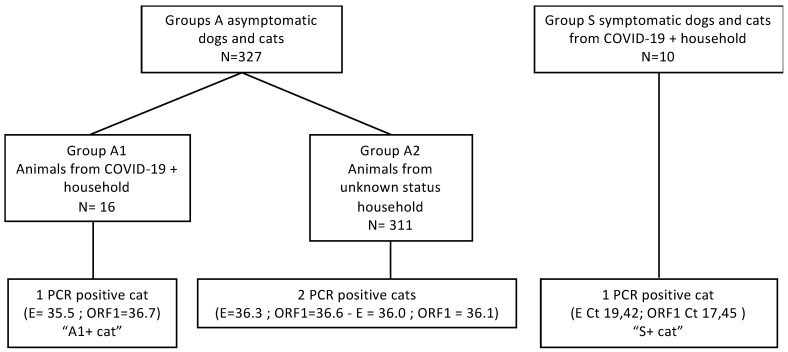
Prospective survey results. Group composition and number of positive animals are displayed. Ct value for targeted genes (E and Orf1) is indicated for positive animals. “A1+ cat” and “S1+ cat” refer to the 2 positive animals with detailed clinical and virological data.

A total of 367 oropharyngeal swabs were analyzed from 337 animals (130 cats, 207 dogs; Figure 1). In Group A, 121 cats and 206 dogs were sampled, while nine cats and one dog were included in Group S. Overall, we identified four positive animals, all of them being cats, and doubtful results (positive for only one gene) were also observed in two animals in A2 (one dog and one cat). 

In detail, one positive cat was found in Group A1, two in Group A2, and one in Group S. Among Group A2 (asymptomatic animals from unknown status households), the two cats were found positive with high Ct values (Ct 35–36). One of the two animals was sampled again 13 days after the initial positive test and was found negative. 

**Figure 2:**
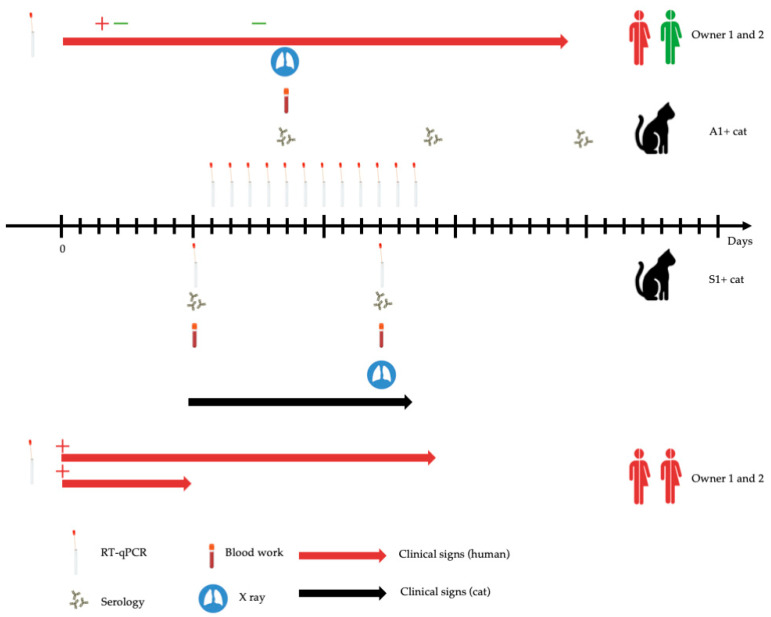
Timeline of the sample collections, clinical exams, and tests in S1+ and A1+ cats. Each household (including animals and their owners) is represented on either side of the timeline. qRT-PCR, serology, blood work, and X-ray pictograms represent the day when the test was performed. For humans, “+” and “−” represent a positive and negative qRT-PCR test, respectively.

The two other positive cases (A1+ cat and S+ cat in Groups A1 and S, respectively) are detailed in the following sections (Figure 2).

### 3.2. A1+ Cat

The first case presented here is a 15-year-old neutered male domestic shorthair cat (A1+ cat) living in an apartment with two persons and kept exclusively indoors (Figure 2). An idiopathic feline lower airways inflammatory disease was suspected in February 2020 based on signs of a chronic cough and abnormal thoracic radiographs, and the cat was treated twice daily with inhaled steroids (fluticasone propionate) therapy. Respiratory signs resolved and the treatment was stopped mid-June 2020. On 1 July 2020, one of the owners (Person A), a 32-year-old man, developed signs of mild respiratory illness including fever and cough, fatigue, and then anosmia. Nasal specimens collected for viral testing on 3 July were positive for SARS-CoV-2 and all the household members were quarantined in their apartment until 15 July. By 26 July, the illness had resolved. The other owner (Person B), a veterinarian, remained healthy and tested negative by qRT-PCR on two occasions (4 and 11 July).

Oropharyngeal swabs were collected on the cat twice daily from 9 July to 21 July, and feces were sampled every time they were passed in order to identify potential SARS-CoV-2 shedding. All samplings were performed by Person B while wearing a face mask and after careful handwashing. The first sample from the cat, obtained 8 days after Person A started showing symptoms, tested positive for SARS-CoV-2 RNA. Further positive results were obtained on 10 and 14 July, always on oropharyngeal samples and with high Ct values ranging from 35.5 to 36.7 (Table 1). Unfortunately, no complete sequencing could be obtained on any samples, probably due to the low level of RNA. Sampling was stopped 7 days after the last positive result. The cat never showed any clinical signs and its demeanor and appetite remained normal. Physical examination performed once a day by Person B never showed any abnormalities. Blood tests (complete blood count, thromboelastometry, and biochemistry including serum electrophoresis and serum amyloid A level) performed on 13 July showed no clinically significant abnormality (Table S1). Thoracic radiographs obtained the same day showed diffuse moderate bronchial and mild interstitial patterns, but the images were considered similar to the ones obtained 5 months previously (Figure S1). Serum samples collected on 13, 21, and 29 July tested negative for SARS-CoV-2 antibodies. 

### 3.3. S+ Cat

**Figure 3:**
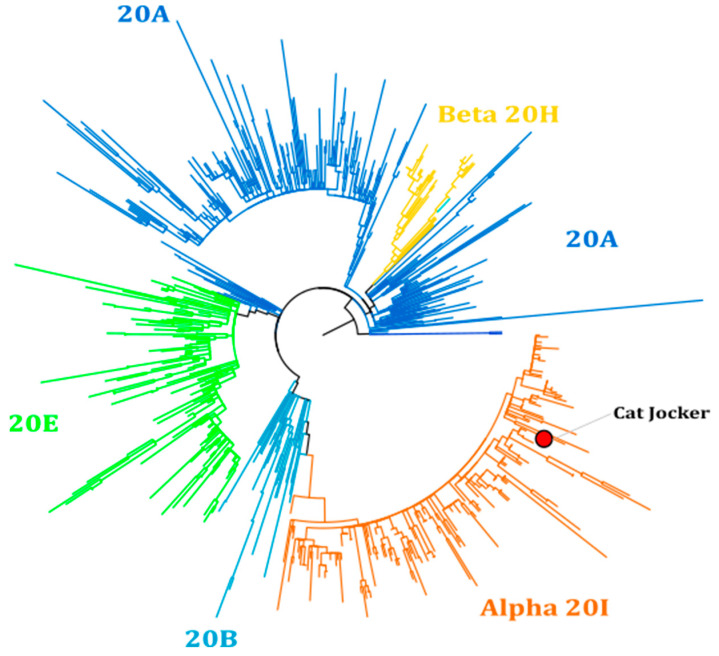
Phylogenic analysis of SARS-CoV-2 genomes, rooted with 4 genomes from the early epidemic and including a representative selection (over 700 genomes) of the diversity of major lineages circulating in France from March 2020 to March 2021.

Another cat, a 1-year-old neutered male domestic shorthair cat (S+ cat) lived in an apartment with two persons with no outdoor access (Figure 2). The usual vaccinations were up to date. The owners tested positive for SARS-CoV-2 one week before. For both owners, Alpha variants were identified and the owners were quarantined in their apartment. On 22 March 2021, the S+ cat was presented to a veterinarian for sneezing without impairment of its general condition. A clinical exam revealed no anomaly (except mild hyperthermia at 39.1 °C) and the S+ cat was discharged with an 8-day treatment of doxycycline (½ tablet of 50 mg SID). Additionally, based on the cat’s clinical signs and the recent COVID-19 infection of the owners, blood, oropharyngeal, and rectal swabs were collected for SARS-CoV-2 testing. SARS-CoV-2 RNA was detected in both the rectal and the oropharyngeal swabs. From the oropharyngeal swab, a Ct of 19.42 for the E gene, and 17.41 for the Orf1 gene was obtained. A lower viral load was obtained from the rectal swab, with Cts of 36.82 and 36.76, respectively (Table 1). The cat was maintained under strict observation and was isolated from other members of the household. Ten days after the initial clinical exam, the S+ cat was presented to a veterinarian for a recheck to look for seroconversion, assess viral excretion, and to monitor biological parameters (Table S1). The clinicopathological exams revealed no anomaly (Figure S2). The oropharyngeal swab was negative for the E and Orf1 genes, but a low viral load could still be detected in the rectal swab (Ct of 39.73 for the E gene). The virology laboratory of Caen University Hospital confirmed the diagnosis and performed the viral RNA sequencing. SARS-CoV-2 genomes were obtained by high-throughput sequencing on RNA extracted from oropharyngeal and rectal swabs. We observed, in both samples, the del3675-3677SGF in Orf1, and del69-70HV and del144Y in spike deletions characterizing the Alpha variant (variant of concern English B1.1.7) (Figure 3). In addition, most mutations usually associated with this VOC were observed in the spike here (A570D, D614G, P681H, T716I, S982A, and D118H) with the exception of A706V. In summary, these genomes provide strong evidence that this cat had been infected by the SARS-CoV-2 Alpha variant and Nextstrain analysis consistently clustered these sequences in the 20I clade (Figure 3). Unfortunately, samples from its symptomatic owners were not available for comparison.

Additionally, serum analysis showed that the S+ cat seroconverted between the first and the second clinical exam. Indeed, antibodies against N, RBD, and tri-S SARS-CoV-2 proteins were detected on day 10, together with robust seroneutralizing activity, while such antibodies and activity were absent from the serum collected on day 0. 

## 4. Discussion

Ever since the beginning of the pandemic and the first reports of companion animal infection, their potential epidemiological role has been debated. Here, we report the results of a prospective survey aimed at better characterizing SARS-CoV-2 shedding in the general companion animal population. Little information is available about the presence of SARS-CoV-2 in the respiratory tract of companion animals in natural conditions. One large-scale study was conducted in Asia, Europe, and North America and failed to identify any SARS-CoV-2 infections in the 4616 samples tested [31]. However, that study relied on samples collected in early 2020, when SARS-CoV-2 circulation was probably lower. This could explain the absence of positive results. Our study took place during a longer period of time, from April 2020 to April 2021 and, therefore, covered all three waves of human infections in the Lyon area of France. Among the 337 animals included in our survey, and despite a relatively high circulation of the virus in the human population, only four animals tested positive, confirming that viral shedding in the companion animals’ population is a low probability event. Three of the animals were in exclusive contact with their owners, and two of them were with a recently infected owner a few days before the cats tested positive for SARS-CoV-2, which strongly suggests human-to-cat transmission consistent with previous findings. One animal was recently adopted and no information was available from its previous environment. For the last positive animal, no additional data were available and it was not possible to sample the animal after the first test. Overall, our findings indicate that SARS-CoV-2 shedding is very rare in asymptomatic animals, suggesting that the risk of viral transmission to humans is probably negligible for the general feline and canine population in this context. However, as a high virus load was identified in one symptomatic cat from a COVID-19 positive household in our study, and also in previous publications [7,32], individual protective measures should be implemented as a precaution when handling an animal coming from a currently or recently positive household.

However, reverse zoonosis toward companion animals seems to be more frequent, as demonstrated by the high prevalence of infection in domestic animals living in COVID-19-positive households [5] and other seroprevalence studies conducted more recently in Europe [6,15]. Overall, most infections seem to be asymptomatic in animals, even though several case reports in different countries have identified symptomatic animals [33,34]. Those reports suffer from an important selection bias since the initial test was motivated by the presence of symptoms without identified etiology.

In our study, none of the animals showed impairment in the clinical condition, with only mild symptoms (sneezing) observed for a few days in one cat. Interestingly, the A1+ cat’s samples showed a high Ct, but the animal remained asymptomatic during the course of the study and did not develop anti-SARS-CoV-2 antibodies (including neutralizing antibodies). Such transient infection without seroconversion has already been observed in humans [35,36] and in cats [37]. However, passive carrying of the virus without infection could not be excluded in this case. Finally, this report describes the first identification of a cat infected with the Alpha variant in France. The infection was associated with a rather low Ct value, suggesting high viral shedding, which could indicate good transmissibility of the Alpha variants in cats, as observed in humans [38,39]. Whether this observation can be generalized would require further investigations and more observations of Alpha-variant infection in companion animals, as only a limited number of cases of Alpha variant infection is available today. The first cases of Alpha variant infection were reported in England [23], and the first complete recovery of the Alpha variant genome sequence in an animal was obtained in Texas, USA [40]. Alpha variants have also been detected in an Italian cat [32] and a dog in Spain [41]. Finally, no Delta variants have been reported in companion animals to date.

## 5. Conclusions

Our results emphasize the need for identifying SARS-CoV-2 in pets and their owners in order to adapt public health recommendations and prevent human-to-animal transmission. It also highlights the importance of collecting more data about new SARS-CoV-2 variants’ transmissibility and pathogenicity in companion animals. 

## Figures and Tables

**Table 1:** SARS-CoV-2 qRT-PCR detection, serology, and seroneutralization on A1+ and S+ cats during the observation period. A complete sequencing of the genome was obtained from the Cat S+ samples (oropharyngeal and rectal) at day 0.

				Days Post Detection													
	Test	Sample	Target	0	1	2	3	4	5	6	7	8	9	10	11	12	20
Cat A1+	qRT-PCR	Oro-pharyngeal	E	35.5	36	-	-	-	36	-	-	-	-	-	-		
			ORF1	36.7	36	-	-	-	36	-	-	-	-	-	-		
		Rectal	E	-	-	-	-	-	-	-	-	-	-	-	-		
			ORF1	-	-	-	-	-	-	-	-	-	-	-	-		
	Serology	Serum	N					-								-	-
			RBD					-								-	-
			S tri					-								-	-
	SNT	Serum	SARS-CoV-2pp					-								-	-
Cat S+	qRT-PCR	Oro-pharyngeal	E	19.4										-			
			ORF1	17.4										-			
		Rectal	E	36.8										39.7			
			ORF1	36.8										-			
	Serology	Serum	N	-										+			
			RBD	-										+			
			S tri	-										+			
	SNT	Serum	SARS-CoV-2pp	-										+			

## Data Availability

SARS-CoV-2 sequences were deposited in GISAID under numbers EPI_ISL_3838696 and EPI_ISL_3857106.

## Supplementary Materials

The following are available online at https://www.mdpi.com/article/10.3390/v13091759/s1, Figure S1: Thoracic radiographs of the A1+ cat: (A) left lateral, (B) right lateral, and (C) dorsoventral views performed on January 2020: moderate diffuse bronchointerstitial pattern consistent with feline lower airways disease; (D) left lateral, (E) right lateral, and (F) dorsoventral views performed on July 2020: images are considered stable from the previous exam; Figure S2: Thoracic radiographs of S+ cat: (A) left lateral and (B) dorsoventral views; Table S1: Hematology and biochemistry exams performed on A1+ and S+ cats.

## Dedication

The authors dedicate this paper to the memory of our colleague and friend Claude Carazzo.
