# Supplementary material for: Report of One-Year Prospective Surveillance of SARS-CoV-2 in Dogs and Cats in France with Various Exposure Risks: Confirmation of a Low Prevalence of Shedding, Detection and Complete Sequencing of an Alpha Variant in a Cat
Source: Viruses. 2021 Sep 3;13(9):1759. doi: 10.3390/v13091759 (PMC8473452; doi:10.3390/v13091759)
Supplement: Supplementary file 1 [file viruses-13-01759-s001.zip › Table S1.pdf]

Table S1 : Hematology and biochemistry exams performed on A1+ and S+ cats

Parameter	Results		Reference interval
	A1+ cat	S+ cat	
WBC (× 10^9/L)	6.3	8.1	5.5–19.5
Lym (× 10^9/L)	0.5 *	4.9	1.2–10.4
Mon (×10^9/L)	0.1	0.1	0.1–0.8
Gran (× 10^9/L)	5.6	2.8	1.4–9.6
RBC (× 10^{12}/L)	9.3	8.7	5–10
HGB (g/dL)	13.3	13.2	8–15
HCT (%)	40.2	43.9	24–45
MCV (μ^3)	43.4	50.2 *	33–48
MCH (pg)	14.4	15.1	13–17
MCHC (g/dL)	33.1	30.1 *	31–36
PLT (x10^9/L)	401	263	230–680
Urea (mmol/L)	9.1	9.3	4–11
Creatinine (μmol/L)	101	125	50–140
ALP (UI/L)	15	13	1–100
ALT (UI/L)	66 *	68 *	16–60
Total bilirubin (μmol/L)	1.5		1–6
Glucose (mmol/L)	4.9		3–8
TP (g/L)	68	69	54–79
Albumin (g/L)	30	31	29–39
Globulins (g/L)	38	39	29–47
SAA (mg/L)	1.6	8.1	0–12
Troponin I (ng/mL)		0.07	0–0.09
EXTEM CT (s)	64 *		34–52
EXTEM CFT (s)	49		41–260
EXTEM α angle (°)	80		59–82
EXTEM MCF (mm)	72		39–75
EXTEM ML (%)	19		0–23

* considered non-significant

Gran, granulocytes; HCT, haematocrit; HGB, haemoglobin; Lym, lymphocytes; MCH, mean corpuscular haemoglobin; MCHC, mean corpuscular haemoglobin concentration; MCV, mean corpuscular volume; Mon, monocytes; MPV, mean platelet volume; PLT, platelets; RBC, red blood cells; WBC, white blood cells; ALP, alkaline phosphatase; ALT, alanine aminotransferase; TP, total proteins; SAA, serum amyloid A; EXTEM, extrinsic pathways rotational thromboelastometry; CT, clotting time; CFT, clot formation time; ML, maximum lysis.
