# Supplementary figures and images for: Report of One-Year Prospective Surveillance of SARS-CoV-2 in Dogs and Cats in France with Various Exposure Risks: Confirmation of a Low Prevalence of Shedding, Detection and Complete Sequencing of an Alpha Variant in a Cat
Source: Viruses. 2021 Sep 3;13(9):1759. doi: 10.3390/v13091759 (PMC8473452; doi:10.3390/v13091759)

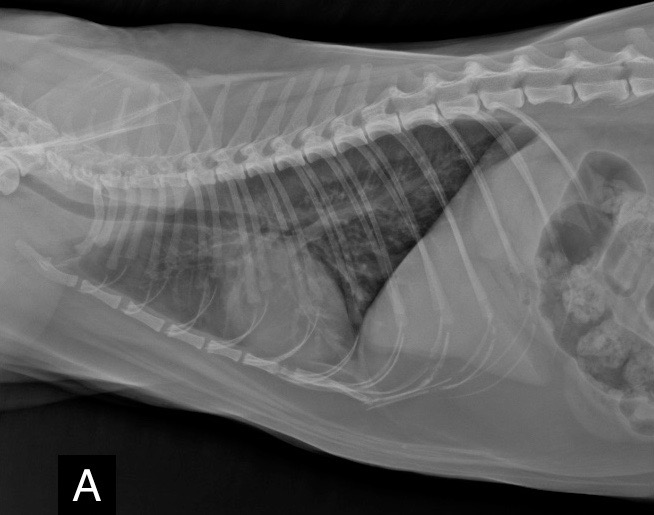

Supplement: Supplementary file 1 [file viruses-13-01759-s001.zip › Figure S1 A.jpeg]

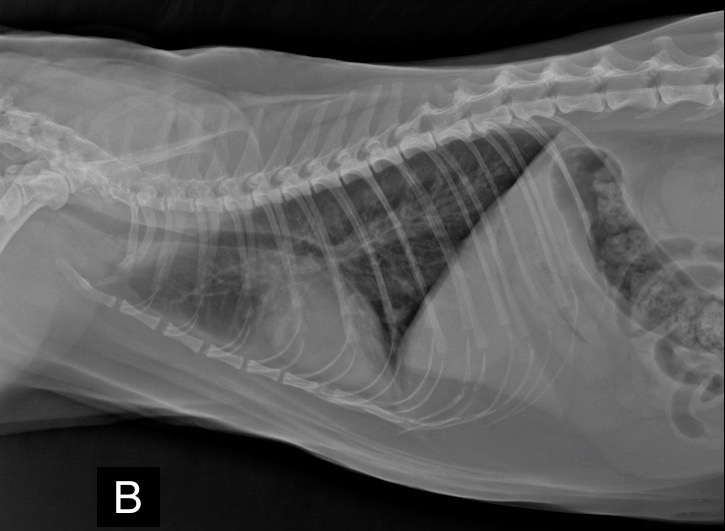

Supplement: Supplementary file 1 [file viruses-13-01759-s001.zip › Figure S1 B.jpeg]

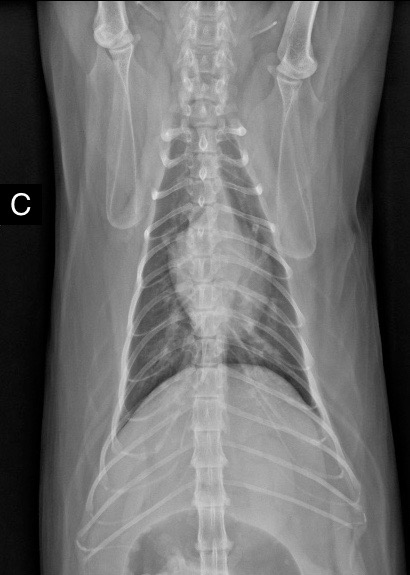

Supplement: Supplementary file 1 [file viruses-13-01759-s001.zip › Figure S1 C.jpeg]

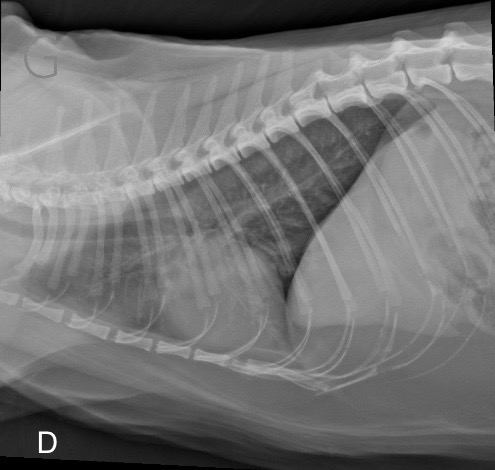

Supplement: Supplementary file 1 [file viruses-13-01759-s001.zip › Figure S1 D.jpeg]

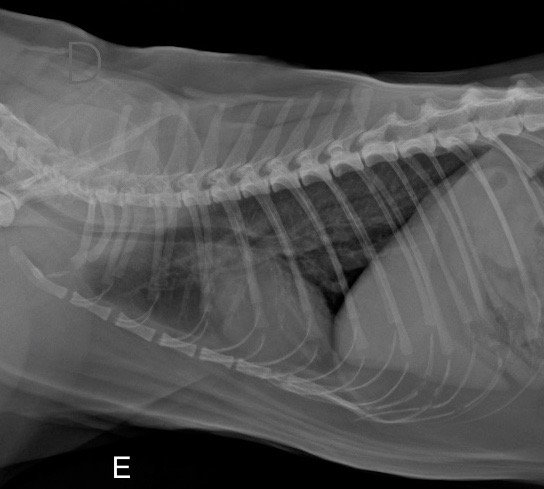

Supplement: Supplementary file 1 [file viruses-13-01759-s001.zip › Figure S1 E.jpeg]

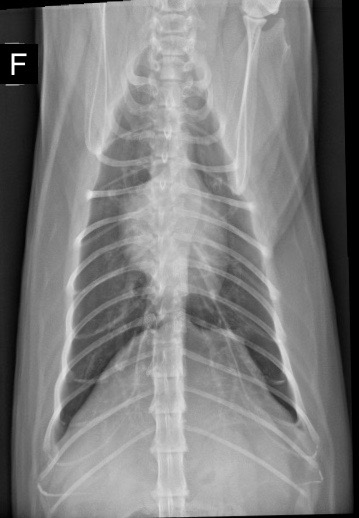

Supplement: Supplementary file 1 [file viruses-13-01759-s001.zip › Figure S1 F.jpeg]

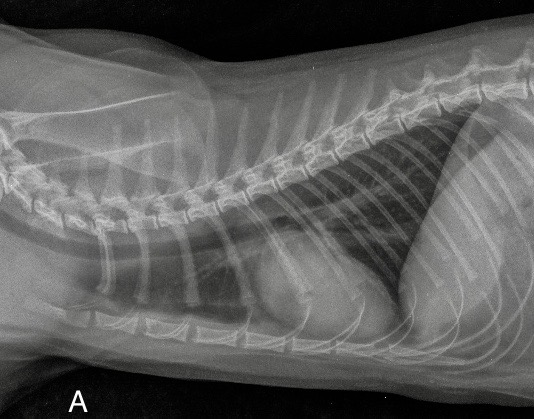

Supplement: Supplementary file 1 [file viruses-13-01759-s001.zip › Figure S2 A.jpeg]

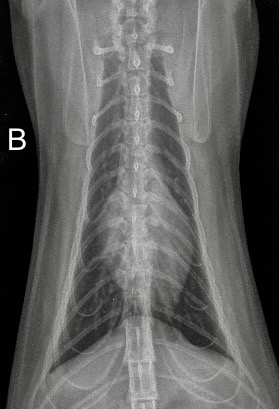

Supplement: Supplementary file 1 [file viruses-13-01759-s001.zip › Figure S2 B.jpeg]
